# Data mobilisation in the LWS Herbarium: success and prospects

**DOI:** 10.3897/BDJ.12.e117292

**Published:** 2024-01-11

**Authors:** Andriy Novikov, Anastasiia Savytska, Oleksandr Kuzyarin, Viktor Nachychko, Solomia Susulovska, Volodymyr Rizun, Andrii Susulovsky, Habriel Hushtan, Kateryna Hushtan, Dmytro Leleka

**Affiliations:** 1 State Museum of Natural History of the NAS of Ukraine, Lviv, Ukraine State Museum of Natural History of the NAS of Ukraine Lviv Ukraine; 2 Ivan Franko National University of Lviv, Lviv, Ukraine Ivan Franko National University of Lviv Lviv Ukraine; 3 Institute of Ecology of the Carpathians of the NAS of Ukraine, Lviv, Ukraine Institute of Ecology of the Carpathians of the NAS of Ukraine Lviv Ukraine

**Keywords:** occurrence, herbarium material, digitisation, Ukraine, flora

## Abstract

**Background:**

Digitisation of hosted specimens is a crucial task for all herbaria worldwide and is one of the main streams for today. By digitising their collections and publishing the datasets, the herbaria grant access to essential data to a wide research audience and, as a result, involve their collections in scientific work more actively. Digitisation also allows virtual preservation of the collections, which is especially important in conditions of hostilities, when the entire collection can be destroyed or damaged in one moment. This paper describes two datasets recently published in GBIF in the framework of the LWS herbarium digitisation initiative. It also contains some considerations about further digitisation priorities and plans in the LWS Herbarium in the context of complicated war conditions and limited facilities.

**New information:**

In total, 2,419 occurrence records from Ukraine mobilised from LWS Herbarium were published. These datasets are planned to be dynamic with the addition of new records along with progress of digitisation work at LWS. At least 6,000 more records are planned to be published through these datasets in 2024.

## Introduction

Herbaria serve as an important source of primary data for many studies, including taxonomic, biogeographic and phylogenetic ones (Holmes et al. 2016, Soltis 2017, James et al. 2018, Ball-Damerow et al. 2019). The herbarium management is a responsible task during which the curators face several issues, including organisation of permanent access, long-term preservation of the collection, unintentional damage to specimens and, occasionally, vandalism (Funk 2002, Pennock 2017, Rabeler et al. 2019). Moreover, processing of the natural history collections, including the herbarium collections, by researchers in person is laborious and expensive (Suarez and Tsutsui 2004, Bradley et al. 2014, Popov et al. 2021). The digitisation and the creation of freely-available datasets and virtual herbaria allow us to solve these issues by making collections quickly and easily accessible through the Internet and providing numerous benefits of remote access (Cantrill 2018, Nieva de la Hidalga et al. 2020, Borsch et al. 2020, Powell et al. 2021, Davis 2023). In light of continuing hostilities in Ukraine (Mosyakin and Shiyan 2022), the mobilisation of biodiversity data and, particularly, the digitisation of herbarium materials acquires a new sense since it could be the only way to preserve, at least virtually, such collections as they are under permanent threat.

The Herbarium of the State Museum of Natural History of the NAS of Ukraine, Lviv (SMNH), is one of the oldest and richest in Ukraine. This Herbarium hosts specimens collected in the Carpathians and other, primarily western, regions of Ukraine. It includes ca. 120,000 specimens of vascular plants and over 26,000 specimens of non-vascular plants and is subdivided into two respective curating units. The commonly-accepted code for both herbarium units is LWS (Thiers 2023).

In late 2023, the State Museum of Natural History of the NAS of Ukraine received a national governmental grant for digitisation of its collections, including herbarium materials. The data about 2419 specimens of vascular and non-vascular plants deposited at LWS were mobilised and published as two datasets (Novikov et al. 2023, Savytska et al. 2023) in the Global Biodiversity Information Facility (GBIF 2023). These datasets will be continually expanded with additional data in the future. At least 6,000 more records are planned to be published through these datasets in 2024.

## General description

### Purpose

The primary purpose of publishing these specimens is to secure the future of the LWS Herbarium, which remains at risk of damage due to hostilities and to make the mobilised data freely and remotely accessible through GBIF. This also aims to improve the implementation of Ukrainian biodiversity data and their integrative use in international research projects.

## Project description

### Title

Digitisation of natural history collections damaged as a result of hostilities and related factors: development of protocols and implementation on the basis of the State Museum of Natural History of the National Academy of Sciences of Ukraine (Nr 2022.01/0013)

### Personnel

**Project PI**: Andriy Novikov (Dr., Senior Research Scientist, SMNH, Department of Biosystematics and Evolution, ORCID https://orcid.org/0000-0002-0112-5070).

**Core Team**: Habriel Hushtan (Dr., Research Scientist, SMNH, Department of Biosystematics and Evolution, ORCID https://orcid.org/0000-0001-6999-6043), Kateryna Hushtan (Dr., Research Scientist, SMNH, Department of Museum Informative Systems, ORCID https://orcid.org/0000-0002-5235-3233), Oleksandr Kuzyarin (Dr., Research Scientist, SMNH, Department of Museum Informative Systems, ORCID https://orcid.org/0000-0002-7728-3665), Bohdan Prots (Dr., Head of Department, SMNH, Department of Landscape and Biotic Diversity, ORCID https://orcid.org/0000-0002-0605-9527), Volodymyr Rizun (Dr., Head of Department, SMNH, Department of Museum Informative Systems, ORCID https://orcid.org/0000-0002-1675-032X), Anastasiia Savytska (Dr., Research Scientist, SMNH, Department of Applied Museology, ORCID https://orcid.org/0000-0002-6255-8590), Andrij Susulovsky (Dr., Head of Department, SMNH, Department of Biosystematics and Evolution, ORCID https://orcid.org/0000-0002-4233-9825).

**Assistants**: Viktor Nachychko (Dr., Associate Professor, Ivan Franko National University of Lviv, Faculty of Biology, Department of Botany, ORCID https://orcid.org/0000-0001-6756-2823), Solomia Susulovska (Dr., Collection Keeper, Junior Research Scientist, Ivan Franko National University of Lviv, Faculty of Biology, Zoological Museum, ORCID https://orcid.org/0000-0001-7585-7584), Dmytro Leleka (PhD Student, Institute of Ecology of the Carpathians of the National Academy of Sciences of Ukraine, Department of Ecosystemology, ORCID https://orcid.org/0000-0002-0112-5070).

### Study area description

The study is mainly focused on the western part of Ukraine, but also includes specimens collected from other regions of the country.

### Design description

The project aims to: (a) develop digitisation protocols for the most valuable and vulnerable natural history collections; (b) mobilise and publish the data about such collections deposited at SMNH; and (c) digitise prioritised specimens deposited at SMNH, including those belonging to the herbarium collection and the collection of invertebrates. The digitisation workflow will include seven stages. During the *Preparatorystage*, the working taxonomic lists are prepared, lists of available materials are synchronised and updated following recent taxonomy, staff roles are ascertained and inventory of the technical equipment is conducted. The *Data Mobilisation* stage includes extraction of the data from the herbarium labels, filling the initial datasets and georeferencing. The *Image Capturing* stage involves preparation of specimens for photography (re-mounting, reparation, label mounting, cleaning etc.), image capturing using the photocameras and image post-processing. The next stage includes the *Synchronisation* of data and images (i.e. renaming the files following the catalogue numbers, ordering images in folders following the applied taxonomy and creating the file by synchronising the links between the images and occurrence records). *Quality Control* is realised continuously during all stages and also involves a final cross-check of the data and digital images. *Archiving* the data and images is realised using internal and external facilities. In particular, the master files and initial datasets are archived on Verbatim MABL BluRay discs and institutional servers. At the same time, the images are archived using Sigma2 AS (2023) and Open Herbarium (2023) platforms. The *Publication* of the data and images is realised using the GBIF (2023), Open Herbarium (2023) and Biodiversity of Ukraine (State Museum of Natural History of the NAS of Ukraine 2023) facilities.

### Funding

The grant programme “Science for the Recovery of Ukraine in the War and Post-War Periods” (Nr 2022.01) of the National Research Foundation of Ukraine (NRFU).

## Sampling methods

### Sampling description

Three priority groups were defined within the LWS Herbarium for digitisation and data mobilisation purposes. The first, red, group comprises type material, authentic collections, specimens of endemic and rare taxa and specimens collected from the 'locus classicus' localities. The second, yellow, group includes specimens of the taxa characteristic for the regional flora and specimens collected from hardly-accessible or currently inaccessible territories (e.g. border territories with limited access and occupied territories). The third, green, group includes specimens of regular species and specimens from supporting (e.g. loan and educational) collections. For the selected taxa, a rough capture of the labels has been conducted. The data from these labels were transferred to the draft tables corresponding to GBIF’s requirements for Occurrence datasets (GBIF 2023). After that, the localities were identified using the OpenStreetMap service and georeferenced. The taxonomy of vascular plants was validated using the GBIF taxonomic backbone (GBIF Secretariat 2023) and cross-checked with POWO (2023). The taxonomy of non-vascular plants was verified using GBIF (2023) taxonomic backbone and also cross-checked with Hodgetts et al. (2020).

### Quality control

The quality of the final datasets was first manually checked. After that, the datasets were processed using OpenRefine 3.7.7 software (OpenRefine 2023) and saved as a tsv file encoded in UTF-8. To test datasets for spatial outliers, QGIS 3.10 software (QGIS Association 2023) has been applied.

### Step description

1. Taking photos of herbarium labels; 2. Re-identification of taxa following recent taxonomy; 3. Extracting the locality, collector, date and other relevant information (e.g. identification history) from the labels; 4. Translation of the primary label information from Slavic languages (i.e. Ukrainian and Russian) to English; 5. Georeferencing of localities using printed maps and OpenStreetMap web service (OpenStreetMap contributors 2023); 6. Quality check applying OpenRefine (OpenRefine 2023) and QGIS (QGIS Association 2023) for detecting outlier and coordinate issues.

## Geographic coverage

### Description

**Figure 1.**
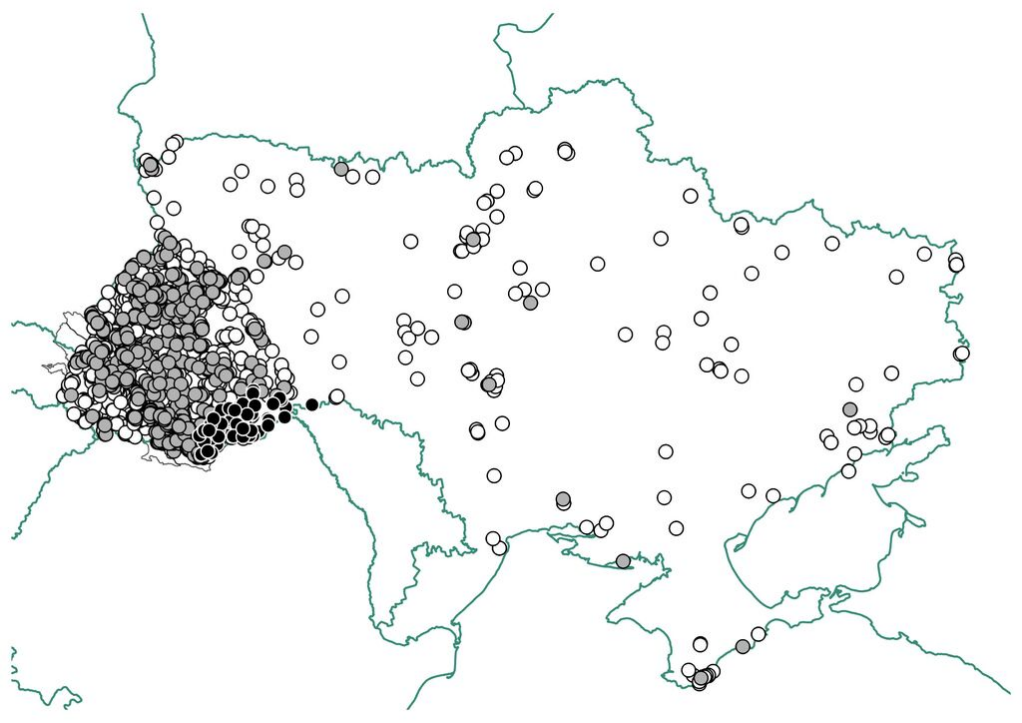
Distribution of occurrences mobilised from the LWS Herbarium in Ukraine. Grey circles indicate occurrences of vascular plants mobilised in 2023; black-filled circles - occurrences of non-vascular plants mobilised in 2023; white circles - occurrences of vascular plants mobilised in 2007-2022.

The data mobilisation in the LWS Herbarium at the moment is focused on Ukrainian flora. Two described datasets containing the data mobilised in 2023 mainly cover the western part of Ukraine, but only occasionally include occurrences from other regions of Ukraine (Fig. 1).

### Coordinates

44.492 and 51.565 Latitude; 22.395 and 37.541 Longitude.

## Taxonomic coverage

### Description

All processed specimens (except two unidentified specimens of Bryales) were identified to the level of species or infraspecies. The dataset of vascular plants contains 59 species, representing 36 genera, 17 families and 11 orders of the class Magnoliopsida (Table 1). Vascular plants' specimens belong mainly to the orders Ericales (28% of total number of processed specimens), Apiales (23%) and Saxifragales (13%). The most abundant families are Apiaceae (23%), Primulaceae (18%) and Ericaceae (10%). The most abundant genera of vascular plants amongst processed specimens are *Primula* (18%) and *Astarntia* (10%). The dataset of non-vascular plants contains 190 species representing 99 genera, 39 families and 11 orders of the class Bryopsida (Table 1). Three of the most represented orders amongst processed specimens of non-vascular plants are Hypnales (57%), Bryales (17%) and Orthotrichales (9%). The most abundant families of non-vascular plants are Brachytheciaceae (15%), Mniaceae (10%), Amblystegiaceae (9%), Orthotrichaceae (9%) and Bryaceae (7%). The genera of non-vascular plants in the processed material are distributed more or less symmetrically.

## Temporal coverage

### Notes

**Figure 2.**
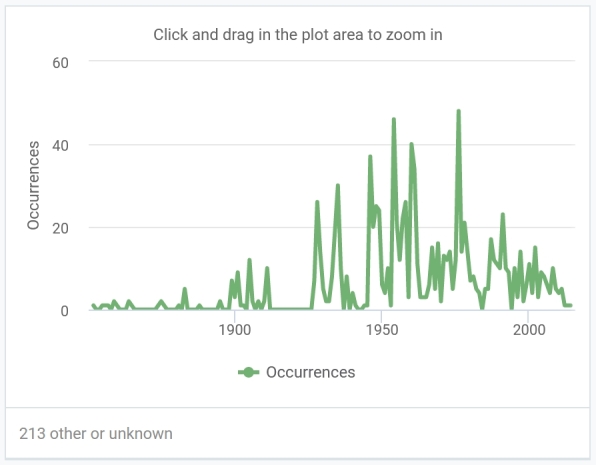
The number of registered occurrences per year in the dataset of vascular plants.

**Figure 3.**
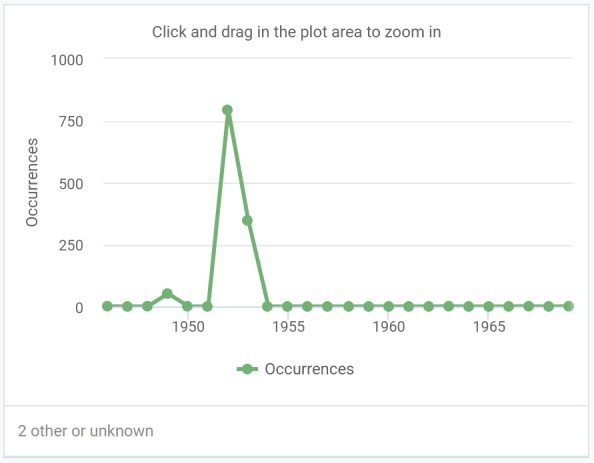
The number of registered occurrences per year in the dataset of non-vascular plants.

The dataset of vascular plants covers specimens collected in 1852–2014 (Fig. 2). The dataset of non-vascular plants covers specimens collected in 1946–1969 (Fig. 3).

## Collection data

### Collection name

Herbarium of the State Museum of Natural History of the NAS of Ukraine.

### Collection identifier

LWS, https://scientific-collections.gbif.org/collection/dd92057d-4fe5-4656-bc5a-90f97456604e

### Specimen preservation method

Dried and pressed.

### Curatorial unit

LWS-vascular and LWS-non-vascular units.

## Usage licence

### Usage licence

Other

### IP rights notes

Creative Commons Attribution License (CC BY 4.0)

## Data resources

### Data package title

LWS Herbarium data mobilisation

### Number of data sets

2

### Data set 1.

#### Data set name

LWS Herbarium. Vascular plants

#### Data format

DarwinCore

#### Character set

UTF-8

#### Download URL

https://doi.org/10.15468/58zxna

#### Description

The tab-delimited CSV-formatted dataset was created following the DarwinCore standard. It contains 1219 occurrence records on the digitised specimens of vascular plants deposited in the LWS Herbarium. This dataset will be dynamically updated with new data along with digitisation and data mobilisation progress in the LWS Herbarium. Currently, it includes the data about 59 species of vascular plants. Currently, this dataset is supported with images of digitised specimens in JPEG format hosted at https://www.sigma2.no/ portal under CC BY 4.0 licence. Further, it will be completed with more data mobilised from LWS, but providing the images will be an option depending on digitisation and hosting facilities.

**Data set 1.** 

Column label	Column description
occurrenceID	An unique identifier for the Occurrence.
basisOfRecord	The specific nature of the data record, for example, preserved specimen or field observation.
institutionCode	The acronym in use by the institution having custody of the object(s) or information referred to in the record.
collectionCode	Unique code of collection (e.g. herbarium) for depositing the identified specimen.
catalogNumber	An identifier for the record within the collection.
scientificName	The full scientific name of the taxon including at least the genus name and species epithet and, in some cases, including the infraspecific epithet.
taxonRank	The taxonomic rank of the most specific name in the scientificName.
kingdom	The full scientific name of the kingdom in which the taxon is classified. In our case, it is always Plantae.
recordedBy	A person, group or organisation responsible for recording the original Occurrence.
verbatimEventDate	The date of record as it appears in the original publication or specimen label.
EventDate	The date during which an event (e.g. collection of the specimen, photographing of the plant or its registering in the field in any other way), occurred.
fieldNumber	An identifier given to the specimen in the field by the collector.
identifiedBy	A list of names of people, who assigned the Taxon to the subject.
dateIdentified	The date on which the subject was determined as representing the Taxon.
identificationRemarks	Comments or notes about the Identification.
decimalLatitude	The geographic latitude (in decimal degrees, using the spatial reference system given in geodeticDatum) of the geographic centre of a Location.
decimalLongitude	The geographic longitude (in decimal degrees, using the spatial reference system given in geodeticDatum) of the geographic centre of a Location.
coordinateUncertaintyInMetres	The horizontal distance (in metres) from the given decimalLatitude and decimalLongitude describing the smallest circle containing the whole of the Location.
geodeticDatum	The ellipsoid, geodetic datum or spatial reference system (SRS), upon which the geographic coordinates given in decimalLatitude and decimalLongitude are based. In our case, it is always WGS84.
minimumElevationInMetres	The lower limit of the range of elevation (altitude, usually above sea level), in metres.
maximumElevationInMetres	The upper limit of the range of elevation (altitude, usually above sea level), in metres.
countryCode	The standard code (ISO 3166-1-alpha-2) for the country in which the Location occurs.
country	The name of the country in which the Location occurs.
locality	The specific description of the place where the specimen was registered or collected.
language	The language of the resource. In our case, herbarium labels contained information in different languages and sometimes different languages were even combined on a single label. To simplify the work with data, we indicated the languages applied for the data.
habitat	The description of the habitat where the specimen was collected or observed.
order	The scientific name of the order.
family	The scientific name of the family.
genus	The scientific name of the genus.
verbatimIdentification	The scientific name under which the specimen is currently stored in the herbarium.
type	The nature or genre of the resource (i.e. StillImage).
format	The format of multimedia file (i.e. image/jpeg).
formulaePath	The field containing the formulae combining hyper-link to the root folder in the hosting resource (i.e. https://storage.gbif-no.sigma2.no/ipt-ukraine-img/2758ae0b-88ac-4e59-8fa8-7f2890317ca0/) with the file names deposited there (file names correspond to catalogNumber).
identifier	The field containing hyper-links to the multimedia files (resulted from the formulaePath field) in a plain text format.
taxon	The Latin name of the species or subspecies related to certain multimedia file (i.e. image).
formulaeTitle	The field containing the formulae combining the phrase "Herbarium specimen image of " with taxon field values.
title	The complete multimedia file (i.e. image) caption (resulted from the formulaeTitle field) in a plain text format.
created	The date of multimedia file (i.e. image) creation.
creator	The name(s) of multimedia file (i.e. image) creator(s).
licence	The licence for the provided multimedia file (i.e. image). In our case, it is CC BY 4.0.
institutionID	An identifier for the institution having custody of the object(s) or information referred to in the record. In our case, we apply ROR IDs.

### Data set 2.

#### Data set name

LWS Herbarium. Non-vascular plants

#### Data format

DarwinCore

#### Character set

UTF-8

#### Download URL

https://doi.org/10.15468/2vyggv

#### Description

The tab-delimited CSV-formatted dataset was created following the DarwinCore standard. It contains 1200 occurrence records on the digitised specimens of non-vascular plants deposited in the LWS Herbarium. This dataset will be dynamically updated with new records according to the digitisation and data mobilisation progress in the LWS Herbarium. Currently, it includes specimens of 190 species of non-vascular plants (bryophyta). It starts with specimens collected and identified by the famous Ukrainian bryologist Kupava Ulychna.

**Data set 2.** 

Column label	Column description
occurrenceID	An unique identifier for the Occurrence.
basisOfRecord	The specific nature of the data record, for example, preserved specimen or field observation.
institutionCode	The acronym in use by the institution having custody of the object(s) or information referred to in the record.
collectionCode	Unique code of collection (e.g. herbarium) for depositing the identified specimen.
catalogNumber	An identifier for the record within the collection.
scientificName	The full scientific name of the taxon including at least the genus name and species epithet and, in some cases, including the subspecies epithet.
taxonRank	The taxonomic rank of the most specific name in the scientificName.
kingdom	The full scientific name of the kingdom in which the taxon is classified. In our case, it is always Plantae.
recordedBy	A person, group or organisation responsible for recording the original Occurrence.
verbatimEventDate	The date of record as it appears in the original publication or specimen label.
EventDate	The date during which an event (e.g. collection of the specimen, photographing of the plant or its registering in the field in any other way) occurred.
fieldNumber	An identifier given to the specimen in the field by the collector.
identifiedBy	A list of names of people, who assigned the Taxon to the subject.
dateIdentified	The date on which the subject was determined as representing the Taxon.
identificationRemarks	Comments or notes about the Identification.
decimalLatitude	The geographic latitude (in decimal degrees, using the spatial reference system given in geodeticDatum) of the geographic centre of a Location.
decimalLongitude	The geographic longitude (in decimal degrees, using the spatial reference system given in geodeticDatum) of the geographic centre of a Location.
coordinateUncertaintyInMetres	The horizontal distance (in metres) from the given decimalLatitude and decimalLongitude describing the smallest circle containing the whole of the Location.
geodeticDatum	The ellipsoid, geodetic datum or spatial reference system (SRS), upon which the geographic coordinates given in decimalLatitude and decimalLongitude are based. In our case, it is always WGS84.
minimumElevationInMetres	The lower limit of the range of elevation (altitude, usually above sea level), in metres.
maximumElevationInMetres	The upper limit of the range of elevation (altitude, usually above sea level), in metres.
countryCode	The standard code (ISO 3166-1-alpha-2) for the country in which the Location occurs.
country	The name of the country in which the Location occurs.
locality	The specific description of the place where the specimen was registered or collected.
language	The language of the resource. In our case, herbarium labels contained information in different languages and sometimes different languages were even combined on a single label. To simplify the work with data, we indicated the languages applied for the data.
habitat	The description of the habitat where the specimen was collected or observed.
order	The scientific name of the order.
family	The scientific name of the family.
genus	The scientific name of the genus.
verbatimIdentification	The scientific name under which the specimen is currently stored in the herbarium.
institutionID	An identifier for the institution having custody of the object(s) or information referred to in the record. In our case, we apply ROR IDs.

## Additional information

### Brief overview of the LWS Herbarium

The State Natural History Museum of the National Academy of Sciences of Ukraine was established as a private collection of the Polish noble family Dzieduszycki (Sas). In 1868, Count Włodzimierz Dzieduszycki bought the current building of the Museum and established the Natural History Museum in Lviv, where he placed his collections. This Museum became gradually open to the public since 1870 and, in 1880, Count Dzieduszycki gifted it to the Polish nation (Petryk 2011, Taborski 2013).

The Herbarium became a valuable part of the Museum’s collections, because it was intensively collected far before its opening, since 1832. Initially, the Herbarium hosted specimens collected personally by Count Dzieduszycki and such famous botanists as Franz Herbich, Ernst Schauer, Antoni Rehmann and Hiacynt Łobarzewski. Later, it was completed by Museum employees Tadeusz Wilczyński, Genadiy Kozij, Jeremi Iwanicki and Fedir Fotyniuk. In the beginning, the Herbarium was entitled “Zielnik Muzeum im. Dzieduszyckich we Lwowie” and registered with the acronym LWD. Later, many collectors complemented it. In particular, in 1940, the Shevchenko Scientific Society in Lviv was liquidated and its collections transferred to the Museum. As a result, the Museum Herbarium has been supplied with specimens collected by Eustach Wołoszczak, Bolesław Kotula, Vinczé von Borbás, Bronisław Błocki, József Barth, Żegota Król, Florian Porcius, Józef Mądalski and many other botanists. Since then, the acronym LWS has been used. In 1950–2000, the Herbarium was intensively supplied with materials collected by Kupava Ulychna, Mykhaylo Slobodian, Kost’ Malynovsiy, Vasyl’ Kolischuk, Anastasiya Lazebna, Ivan Vaynagiy, Vasyl’ Tkachyk, Lydia Tasenkevich, Oleksandr Kuzyarin and other Ukrainian botanists. In general, there are ca. 500 collectors who contributed to the LWS Herbarium. The oldest specimens deposited in the LWS Herbarium were collected by Ernst Wittmann between 1807 and 1811 (Tasenkevich et al. 2009, Tasenkevich and Danyliuk 2011).

The LWS Herbarium currently hosts ca. 120,000 specimens of vascular plants and over 26,000 specimens of non-vascular plants. Amongst them, ca. 200 specimens represent type material, but this number is approximate since the typification is still unfinished. Additionally, it includes ca. 4,000 specimens deposited in supporting (loan and educational) collections.

Most of the deposited specimens are collected from the local flora, with emphasis on the western part of Ukraine (i.e. Lviv, Ivano-Frankivsk, Ternopil, Chernivtsi, Zakarpattia and Volhynia oblasts). However, LWS also hosts many specimens collected from the countries of the Carpathian region, i.e. Poland, Slovakia, Czech Republic, Romania and Hungary.

### History and prospects of digitisation and data mobilisation in the LWS Herbarium

Digitisation of the LWS Herbarium began in 2012–2013 when type specimens of vascular plants were digitised in the context of a Mellon Foundation grant. In particular, the data and images of 286 specimens, including 252 type specimens of vascular plants, were deposited at JACQ (JACQ consortium 2023) and JSTOR Global Plants (ITHAKA 2023). In 2007–2020, the data on the genus *Aconitum* were mobilised and 420 records from the LWS Herbarium were added to this dataset (Novikov and Prylutskyi 2023). In 2021–2022, funded by an IAPT Small Collections grant, the data about 1,873 specimens of vascular plants deposited in the LWS Herbarium were mobilised. These records supplemented the investigations of endemism in the flora of the Ukrainian Carpathians and were published within the respective dataset (Novikov and Sup-Novikova 2022). In 2023, this dataset has been updated with additional georeferenced data and supported by images. In 2022–2023, 11,437 more occurrence records were mobilised from LWS and published through GBIF. This dataset comprised rare, relict, range-limited and problematic taxa of vascular plants in the Ukrainian Carpathians and adjacent territories (Novikov 2023). In 2023, 1,219 specimens of vascular plants were digitised within an NRFU grant and respective data were published to GBIF (Novikov et al. 2023) and Open Herbarium (SMNH-NASU-LWS 2023) platforms. Hence, at the moment, the data regarding 15,235 LWS’s specimens of vascular plants (12.7% of the total number of deposited specimens) have been mobilised and published online. In particular, 3092 specimens of vascular plants (2.6%) have been digitised, and their images appeared online in JACQ (JACQ consortium 2023), GBIF (2023) and Open Herbarium (2023) platforms. The digitisation of the Herbarium of non-vascular plants started only in 2023 and, at the moment, only 1200 specimens (4.6%) have been digitised. Their images are available through the Biodiversity of Ukraine (State Museum of Natural History of the NAS of Ukraine 2023) database. In 2024, with NRFU support, we are going to digitise ca. 5,000 specimens of vascular plants (+4.2%) and ca. 2,000 specimens of non-vascular plants (+7.7%). The data mobilised during this work will be published as updates to the datasets described here. High-resolution (80 Mp) specimens' images from the herbarium of vascular plants and middle-resolution (20 Mp) specimen images from the herbarium of non-vascular plants will be made available online through GBIF (2023) and Open Herbarium (2023) platforms and other online resources.

### Digitisation priorities for the near future

The current digitisation in the LWS Herbarium is focused on the red priority group, limited by the flora of Ukraine. From this group, the specimens of the type material have already been digitised and are available online through the JACQ (JACQ consortium 2023) and JSTOR Global Plants (ITHAKA 2023) portals. The digitisation of specimens of endemic and rare taxa and authentic collections is currently in progress. The next digitisation round (2025–2030) will involve the specimens belonging to the yellow priority group (i.e. taxa characterising the regional flora and hardly-accessible or currently inaccessible territories). The specimens of the green priority group, belonging to the trivial flora, will be digitised after that.

Specimens collected from other countries are not part of current digitisation plans and will be digitised later or on request. This geographic scope, limited by the Ukrainian borders, has been ascertained due to several reasons: (a) limited financial and technical facilities available for digitisation ; (b) floras of other countries are rather in response of respective herbaria and can be covered by such herbaria, while they cannot successfully cover the flora of Ukraine; (c) absence of expertise in other floras limits the success of resolving taxonomic issues that could arise during the digitisation of materials collected out of Ukraine; (d) limited expertise in toponymy of other countries can result in multiple incorrect data extraction and/or georeferencing and significantly inhibits the digitisation process.

We will gladly consider requests for prioritised digitisation from scientists worldwide. We believe that it is most important to digitise those materials that are urgently needed for research purposes. Therefore, please direct your requests, including the list of taxa and brief explanation for your request, to the herbarium curators, Andriy Novikov (novikoffav@gmail.com) and Anastasiia Savytska (asavitska@gmail.com).

## Figures and Tables

**Table 1.** The list of taxa and occurrences mobilised from the LWS herbarium in 2023.

Order	Family	Genus	Species	Occurrences
Apiales	Apiaceae	*Astrantia*	*Astrantiamajor*	121
Apiales	Apiaceae	*Bupleurum*	*Bupleurumfalcatum*	66
Apiales	Apiaceae	*Bupleurum*	*Bupleurumlongifolium*	13
Apiales	Apiaceae	*Bupleurum*	*Bupleurumtenuissimum*	1
Apiales	Apiaceae	*Meum*	*Meumathamanticum*	1
Apiales	Apiaceae	*Pleurospermum*	*Pleurospermumaustriacum*	34
Apiales	Apiaceae	*Seseli*	*Seselilibanotis*	41
Caryophyllales	Caryophyllaceae	*Dianthus*	*Dianthuscarthusianorum*	11
Caryophyllales	Caryophyllaceae	*Dianthus*	*Dianthussuperbus*	23
Caryophyllales	Caryophyllaceae	*Dianthus*	*Dianthussuperbus*	1
Caryophyllales	Caryophyllaceae	*Dianthus*	*Dianthustrifasciculatus*	1
Crossosomatales	Staphyleaceae	*Staphylea*	*Staphyleapinnata*	38
Ericales	Ericaceae	*Kalmia*	*Kalmiaprocumbens*	22
Ericales	Ericaceae	*Rhododendron*	*Rhododendronkotschyi*	88
Ericales	Ericaceae	*Vaccinium*	*Vacciniummicrocarpum*	11
Ericales	Primulaceae	*Primula*	*Primulaelatior*	166
Ericales	Primulaceae	*Primula*	*Primulahalleri*	14
Ericales	Primulaceae	*Primula*	*Primulamatthioli*	19
Ericales	Primulaceae	*Primula*	*Primulaminima*	21
Fabales	Fabaceae	*Anthyllis*	*Anthyllisvulneraria*	4
Fabales	Fabaceae	*Astragalus*	*Astragalusaustralis*	1
Fabales	Fabaceae	*Chamaecytisus*	*Chamaecytisusalbus*	34
Fabales	Fabaceae	*Chamaecytisus*	*Chamaecytisuslindemannii*	1
Fabales	Fabaceae	*Chamaecytisus*	*Chamaecytisuspodolicus*	2
Fabales	Fabaceae	*Coronilla*	*Coronillaelegans*	5
Fabales	Fabaceae	*Cytisus*	*Cytisuskerneri*	1
Fabales	Fabaceae	*Genista*	*Genistasagittalis*	5
Fabales	Fabaceae	*Lathyrus*	*Lathyruslaevigatus*	24
Fabales	Fabaceae	*Trifolium*	*Trifoliumpratense*	1
Fabales	Fabaceae	*Trifolium*	*Trifoliumrubens*	14
Gentianales	Gentianaceae	*Gentiana*	*Gentianaacaulis*	16
Gentianales	Gentianaceae	*Gentiana*	*Gentianalutea*	15
Gentianales	Gentianaceae	*Gentiana*	*Gentiananivalis*	1
Gentianales	Gentianaceae	*Gentiana*	*Gentianapunctata*	29
Gentianales	Gentianaceae	*Gentiana*	*Gentianaverna*	1
Gentianales	Gentianaceae	*Gentianopsis*	*Gentianopsisciliata*	22
Gentianales	Gentianaceae	*Swertia*	*Swertiaperennis*	24
Lamiales	Oleaceae	*Fraxinus*	*Fraxinusornus*	1
Malpighiales	Euphorbiaceae	*Euphorbia*	*Euphorbiacarpatica*	7
Malpighiales	Euphorbiaceae	*Euphorbia*	*Euphorbiadulcis*	1
Malpighiales	Euphorbiaceae	*Euphorbia*	*Euphorbiaillirica*	9
Malpighiales	Hypericaceae	*Hypericum*	*Hypericumricheri*	48
Malpighiales	Violaceae	*Viola*	*Violaalba*	4
Malpighiales	Violaceae	*Viola*	*Violadacica*	6
Malpighiales	Violaceae	*Viola*	*Violatricolor*	4
Malvales	Cistaceae	*Helianthemum*	*Helianthemumnummularium*	2
Malvales	Cistaceae	*Helianthemum*	*Helianthemumnummularium*	17
Malvales	Malvaceae	*Tilia*	*Tiliaplatyphyllos*	8
Rosales	Rosaceae	*Alchemilla*	*Alchemillaszaferi*	3
Rosales	Rosaceae	*Comarum*	*Comarumpalustre*	36
Rosales	Rosaceae	*Dryas*	*Dryasoctopetala*	9
Rosales	Rosaceae	*Rosa*	*Rosagallica*	12
Rosales	Rosaceae	*Torminalis*	*Torminalisglaberrima*	4
Saxifragales	Crassulaceae	*Rhodiola*	*Rhodiolarosea*	44
Saxifragales	Grossulariaceae	*Ribes*	*Ribespetraeum*	13
Saxifragales	Saxifragaceae	*Saxifraga*	*Saxifragaadscendens*	4
Saxifragales	Saxifragaceae	*Saxifraga*	*Saxifragaaizoides*	7
Saxifragales	Saxifragaceae	*Saxifraga*	*Saxifragaandrosacea*	3
Saxifragales	Saxifragaceae	*Saxifraga*	*Saxifragabryoides*	13
Saxifragales	Saxifragaceae	*Saxifraga*	*Saxifragacarpatica*	1
Saxifragales	Saxifragaceae	*Saxifraga*	*Saxifragapaniculata*	71
Aulacomniales	Aulacomniaceae	*Aulacomnium*	*Aulacomniumpalustre*	4
Bartramiales	Bartramiaceae	*Bartramia*	*Bartramiahalleriana*	6
Bartramiales	Bartramiaceae	*Bartramia*	*Bartramiaithyphylla*	3
Bartramiales	Bartramiaceae	*Philonotis*	*Philonotiscaespitosa*	1
Bartramiales	Bartramiaceae	*Philonotis*	*Philonotiscalcarea*	1
Bartramiales	Bartramiaceae	*Philonotis*	*Philonotisfontana*	1
Bartramiales	Bartramiaceae	*Philonotis*	*Philonotismarchica*	1
Bryales	Bryaceae	*Bryum*	*Bryumargenteum*	5
Bryales	Bryaceae	*Bryum*	*Bryumbicolor*	2
Bryales	Bryaceae	*Bryum*	*Bryuminclinatum*	1
Bryales	Bryaceae	*Bryum*	*Bryumlanatum*	12
Bryales	Bryaceae	*Gemmabryum*	*Gemmabryumbadium*	1
Bryales	Bryaceae	*Gemmabryum*	*Gemmabryumcaespiticium*	9
Bryales	Bryaceae	*Gemmabryum*	*Gemmabryumklinggraeffii*	1
Bryales	Bryaceae	*Ptychostomum*	*Ptychostomumbimum*	5
Bryales	Bryaceae	*Ptychostomum*	*Ptychostomumcernuum*	1
Bryales	Bryaceae	*Ptychostomum*	*Ptychostomumcompactum*	1
Bryales	Bryaceae	*Ptychostomum*	*Ptychostomumpallescens*	1
Bryales	Bryaceae	*Ptychostomum*	*Ptychostomumpseudotriquetrum*	7
Bryales	Bryaceae	*Ptychostomum*	*Ptychostomumturbinatum*	4
Bryales	Bryaceae	*Rosulabryum*	*Rosulabryumcapillare*	15
Bryales	Bryaceae	*Rosulabryum*	*Rosulabryummoravicum*	13
Bryales	Bryaceae	*Rosulabryum*	*Rosulabryumrubens*	4
Bryales	Mniaceae	*Mnium*	*Mniumhornum*	1
Bryales	Mniaceae	*Mnium*	*Mniummarginatum*	11
Bryales	Mniaceae	*Mnium*	*Mniumspinosum*	2
Bryales	Mniaceae	*Mnium*	*Mniumstellare*	11
Bryales	Mniaceae	*Mnium*	*Mniumthomsonii*	2
Bryales	Mniaceae	*Plagiomnium*	*Plagiomniumcuspidatum*	22
Bryales	Mniaceae	*Plagiomnium*	*Plagiomniumelatum*	10
Bryales	Mniaceae	*Plagiomnium*	*Plagiomniumellipticum*	2
Bryales	Mniaceae	*Plagiomnium*	*Plagiomniumrostratum*	11
Bryales	Mniaceae	*Plagiomnium*	*Plagiomniumundulatum*	23
Bryales	Mniaceae	*Pohlia*	*Pohliacruda*	1
Bryales	Mniaceae	*Pohlia*	*Pohliaelongata*	3
Bryales	Mniaceae	*Pohlia*	*Pohliafilum*	1
Bryales	Mniaceae	*Pohlia*	*Pohlialongicolla*	2
Bryales	Mniaceae	*Pohlia*	*Pohlianutans*	5
Bryales	Mniaceae	*Rhizomnium*	*Rhizomniumpunctatum*	11
Dicranales	Dicranaceae	*Dicranum*	*Dicranumbonjeanii*	5
Dicranales	Dicranaceae	*Dicranum*	*Dicranumfuscescens*	3
Dicranales	Dicranaceae	*Dicranum*	*Dicranumpolysetum*	5
Dicranales	Dicranaceae	*Dicranum*	*Dicranumscoparium*	1
Dicranales	Dicranaceae	*Dicranum*	*Dicranumviride*	5
Dicranales	Dicranaceae	*Orthodicranum*	*Orthodicranummontanum*	15
Dicranales	Dicranaceae	*Paraleucobryum*	*Paraleucobryumlongifolium*	4
Dicranales	Ditrichaceae	*Ditrichum*	*Ditrichumheteromallum*	3
Dicranales	Ditrichaceae	*Ditrichum*	*Ditrichumpallidum*	3
Dicranales	Ditrichaceae	*Ditrichum*	*Ditrichumpusillum*	2
Dicranales	Ditrichaceae	*Pleuridium*	*Pleuridiumsubulatum*	5
Dicranales	Fissidentaceae	*Fissidens*	*Fissidensadianthoides*	2
Dicranales	Fissidentaceae	*Fissidens*	*Fissidensbryoides*	10
Dicranales	Fissidentaceae	*Fissidens*	*Fissidenscrispus*	2
Dicranales	Fissidentaceae	*Fissidens*	*Fissidensgymnandrus*	1
Dicranales	Fissidentaceae	*Fissidens*	*Fissidensobtusifolius*	1
Dicranales	Fissidentaceae	*Fissidens*	*Fissidenspusillus*	3
Dicranales	Fissidentaceae	*Fissidens*	*Fissidensrufescens*	1
Dicranales	Fissidentaceae	*Fissidens*	*Fissidensviridulus*	2
Dicranales	Rhabdoweisiaceae	*Dicranoweisia*	*Dicranoweisiacirrata*	1
Encalyptales	Encalyptaceae	*Encalypta*	*Encalyptaciliata*	2
Encalyptales	Encalyptaceae	*Encalypta*	*Encalyptastreptocarpa*	4
Encalyptales	Encalyptaceae	*Encalypta*	*Encalyptavulgaris*	1
Funariales	Funariaceae	*Entosthodon*	*Entosthodonhungaricus*	3
Funariales	Funariaceae	*Entosthodon*	*Entosthodonhungaricus*	8
Funariales	Funariaceae	*Funaria*	*Funariahygrometrica*	16
Funariales	Funariaceae	*Physcomitrium*	*Physcomitriumacuminatum*	1
Funariales	Funariaceae	*Physcomitrium*	*Physcomitriumeurystomum*	2
Funariales	Funariaceae	*Physcomitrium*	*Physcomitriumpatens*	1
Funariales	Funariaceae	*Physcomitrium*	*Physcomitriumpyriforme*	4
Grimmiales	Grimmiaceae	*Coscinodon*	*Coscinodoncribrosus*	1
Grimmiales	Grimmiaceae	*Grimmia*	*Grimmiapulvinata*	1
Grimmiales	Grimmiaceae	*Niphotrichum*	*Niphotrichumcanescens*	2
Grimmiales	Grimmiaceae	*Schistidium*	*Schistidiumapocarpum*	1
Grimmiales	Ptychomitriaceae	*Campylostelium*	*Campylosteliumsaxicola*	2
Grimmiales	Seligeriaceae	*Blindia*	*Blindiaacuta*	9
Grimmiales	Seligeriaceae	*Blindiadelphus*	*Blindiadelphusrecurvatus*	8
Hypnales	Amblystegiaceae	*Amblystegium*	*Amblystegiumserpens*	36
Hypnales	Amblystegiaceae	*Anacamptodon*	*Anacamptodonsplachnoides*	2
Hypnales	Amblystegiaceae	*Campylium*	*Campyliumchrysophyllum*	8
Hypnales	Amblystegiaceae	*Campylium*	*Campyliumstellatum*	15
Hypnales	Amblystegiaceae	*Campylophyllopsis*	*Campylophyllopsissommerfeltii*	6
Hypnales	Amblystegiaceae	*Conardia*	*Conardiacompacta*	2
Hypnales	Amblystegiaceae	*Drepanium*	*Drepaniumfastigiatum*	1
Hypnales	Amblystegiaceae	*Drepanocladus*	*Drepanocladuspolygamus*	3
Hypnales	Amblystegiaceae	*Drepanocladus*	*Drepanocladuspolygamus*	1
Hypnales	Amblystegiaceae	*Hygroamblystegium*	*Hygroamblystegiumtenax*	1
Hypnales	Amblystegiaceae	*Hygroamblystegium*	*Hygroamblystegiumvarium*	3
Hypnales	Amblystegiaceae	*Hygrohypnum*	*Hygrohypnumluridum*	14
Hypnales	Amblystegiaceae	*Leptodictyum*	*Leptodictyumriparium*	1
Hypnales	Amblystegiaceae	*Pseudoamblystegium*	*Pseudoamblystegiumsubtile*	10
Hypnales	Amblystegiaceae	*Serpoleskea*	*Serpoleskeaconfervoides*	4
Hypnales	Amblystegiaceae	*Tomentypnum*	*Tomentypnumnitens*	1
Hypnales	Anomodontaceae	*Anomodon*	*Anomodonviticulosus*	10
Hypnales	Anomodontaceae	*Anomodontella*	*Anomodontellalongifolia*	2
Hypnales	Anomodontaceae	*Anomodontopsis*	*Anomodontopsisrugelii*	1
Hypnales	Brachytheciaceae	*Brachytheciastrum*	*Brachytheciastrumvelutinum*	16
Hypnales	Brachytheciaceae	*Brachythecium*	*Brachytheciumalbicans*	7
Hypnales	Brachytheciaceae	*Brachythecium*	*Brachytheciumcampestre*	8
Hypnales	Brachytheciaceae	*Brachythecium*	*Brachytheciumglareosum*	6
Hypnales	Brachytheciaceae	*Brachythecium*	*Brachytheciummildeanum*	3
Hypnales	Brachytheciaceae	*Brachythecium*	*Brachytheciumrivulare*	5
Hypnales	Brachytheciaceae	*Brachythecium*	*Brachytheciumrutabulum*	24
Hypnales	Brachytheciaceae	*Brachythecium*	*Brachytheciumsalebrosum*	15
Hypnales	Brachytheciaceae	*Cirriphyllum*	*Cirriphyllumpiliferum*	14
Hypnales	Brachytheciaceae	*Eurhynchiastrum*	*Eurhynchiastrumpulchellum*	1
Hypnales	Brachytheciaceae	*Eurhynchium*	*Eurhynchiumangustirete*	17
Hypnales	Brachytheciaceae	*Eurhynchium*	*Eurhynchiumstriatum*	3
Hypnales	Brachytheciaceae	*Homalothecium*	*Homalotheciumlutescens*	2
Hypnales	Brachytheciaceae	*Kindbergia*	*Kindbergiapraelonga*	3
Hypnales	Brachytheciaceae	*Oxyrrhynchium*	*Oxyrrhynchiumhians*	27
Hypnales	Brachytheciaceae	*Pseudoscleropodium*	*Pseudoscleropodiumpurum*	2
Hypnales	Brachytheciaceae	*Rhynchostegium*	*Rhynchostegiummurale*	8
Hypnales	Brachytheciaceae	*Rhynchostegium*	*Rhynchostegiumriparioides*	13
Hypnales	Brachytheciaceae	*Sciuro-hypnum*	*Sciuro-hypnum plumosum*	1
Hypnales	Brachytheciaceae	*Sciuro-hypnum*	*Sciuro-hypnum populeum*	8
Hypnales	Calliergonaceae	*Calliergon*	*Calliergoncordifolium*	1
Hypnales	Calliergonaceae	*Sarmentypnum*	*Sarmentypnumexannulatum*	1
Hypnales	Climaciaceae	*Climacium*	*Climaciumdendroides*	24
Hypnales	Entodontaceae	*Entodon*	*Entodonconcinnus*	2
Hypnales	Fontinalaceae	*Fontinalis*	*Fontinalisantipyretica*	2
Hypnales	Hylocomiaceae	*Hylocomiadelphus*	*Hylocomiadelphustriquetrus*	2
Hypnales	Hylocomiaceae	*Rhytidiadelphus*	*Rhytidiadelphussquarrosus*	9
Hypnales	Hylocomiaceae	*Rhytidiadelphus*	*Rhytidiadelphussubpinnatus*	2
Hypnales	Hypnaceae	*Hypnum*	*Hypnumcupressiforme*	24
Hypnales	Hypnaceae	*Hypnum*	*Hypnumcupressiforme*	4
Hypnales	Jocheniaceae	*Jochenia*	*Jocheniapallescens*	5
Hypnales	Lembophyllaceae	*Isothecium*	*Isotheciumalopecuroides*	16
Hypnales	Leskeaceae	*Leskea*	*Leskeapolycarpa*	19
Hypnales	Leucodontaceae	*Leucodon*	*Leucodonsciuroides*	29
Hypnales	Myuriaceae	*Ctenidium*	*Ctenidium molluscum*	10
Hypnales	Neckeraceae	*Alleniella*	*Alleniellacomplanata*	4
Hypnales	Neckeraceae	*Homalia*	*Homaliatrichomanoides*	9
Hypnales	Neckeraceae	*Pseudanomodon*	*Pseudanomodonattenuatus*	13
Hypnales	Plagiotheciaceae	*Herzogiella*	*Herzogiellaseligeri*	10
Hypnales	Plagiotheciaceae	*Orthothecium*	*Orthotheciumintricatum*	4
Hypnales	Plagiotheciaceae	*Plagiothecium*	*Plagiotheciumcavifolium*	9
Hypnales	Plagiotheciaceae	*Plagiothecium*	*Plagiotheciumdenticulatum*	2
Hypnales	Plagiotheciaceae	*Plagiothecium*	*Plagiotheciumdenticulatum*	1
Hypnales	Plagiotheciaceae	*Plagiothecium*	*Plagiotheciumlaetum*	1
Hypnales	Plagiotheciaceae	*Plagiothecium*	*Plagiotheciumnemorale*	9
Hypnales	Plagiotheciaceae	*Plagiothecium*	*Plagiotheciumplatyphyllum*	3
Hypnales	Plagiotheciaceae	*Plagiothecium*	*Plagiotheciumsucculentum*	1
Hypnales	Plagiotheciaceae	*Plagiothecium*	*Plagiotheciumundulatum*	1
Hypnales	Pseudoleskeellaceae	*Pseudoleskeella*	*Pseudoleskeellanervosa*	9
Hypnales	Pterigynandraceae	*Pterigynandrum*	*Pterigynandrumfiliforme*	3
Hypnales	Pylaisiaceae	*Buckia*	*Buckiavaucheri*	2
Hypnales	Pylaisiaceae	*Calliergonella*	*Calliergonellacuspidata*	29
Hypnales	Pylaisiaceae	*Calliergonella*	*Calliergonellalindbergii*	1
Hypnales	Pylaisiaceae	*Homomallium*	*Homomalliumincurvatum*	9
Hypnales	Pylaisiaceae	*Ptilium*	*Ptiliumcrista-castrensis*	6
Hypnales	Pylaisiaceae	*Pylaisia*	*Pylaisiapolyantha*	23
Hypnales	Pylaisiadelphaceae	*Platygyrium*	*Platygyriumrepens*	4
Hypnales	Rhytidiaceae	*Rhytidium*	*Rhytidiumrugosum*	1
Hypnales	Scorpidiaceae	*Sanionia*	*Sanioniauncinata*	6
Hypnales	Taxiphyllaceae	*Taxiphyllum*	*Taxiphyllumwissgrillii*	5
Hypnales	Thuidiaceae	*Abietinella*	*Abietinellaabietina*	21
Hypnales	Thuidiaceae	*Thuidium*	*Thuidiumassimile*	25
Hypnales	Thuidiaceae	*Thuidium*	*Thuidiumdelicatulum*	1
Hypnales	Thuidiaceae	*Thuidium*	*Thuidiumrecognitum*	6
Hypnales	Thuidiaceae	*Thuidium*	*Thuidiumtamariscinum*	7
Orthotrichales	Orthotrichaceae	*Lewinskya*	*Lewinskyaaffinis*	2
Orthotrichales	Orthotrichaceae	*Lewinskya*	*Lewinskyafastigiata*	6
Orthotrichales	Orthotrichaceae	*Lewinskya*	*Lewinskyaspeciosa*	19
Orthotrichales	Orthotrichaceae	*Lewinskya*	*Lewinskyastriata*	8
Orthotrichales	Orthotrichaceae	*Nyholmiella*	*Nyholmiellaobtusifolia*	14
Orthotrichales	Orthotrichaceae	*Orthotrichum*	*Orthotrichumanomalum*	13
Orthotrichales	Orthotrichaceae	*Orthotrichum*	*Orthotrichumcupulatum*	2
Orthotrichales	Orthotrichaceae	*Orthotrichum*	*Orthotrichumpallens*	6
Orthotrichales	Orthotrichaceae	*Orthotrichum*	*Orthotrichumpatens*	5
Orthotrichales	Orthotrichaceae	*Orthotrichum*	*Orthotrichumpumilum*	12
Orthotrichales	Orthotrichaceae	*Orthotrichum*	*Orthotrichumscanicum*	1
Orthotrichales	Orthotrichaceae	*Orthotrichum*	*Orthotrichumstramineum*	2
Orthotrichales	Orthotrichaceae	*Orthotrichum*	*Orthotrichumtenellum*	1
Orthotrichales	Orthotrichaceae	*Pulvigera*	*Pulvigeralyellii*	1
Orthotrichales	Orthotrichaceae	*Ulota*	*Ulotacoarctata*	1
Orthotrichales	Orthotrichaceae	*Ulota*	*Ulotacrispa*	6
Orthotrichales	Orthotrichaceae	*Ulota*	*Ulotacrispula*	5
Orthotrichales	Orthotrichaceae	*Ulota*	*Ulotahutchinsiae*	2
Pottiales	Pottiaceae	*Bryoerythrophyllum*	*Bryoerythrophyllumrecurvirostrum*	4
Pottiales	Pottiaceae	*Eucladium*	*Eucladiumverticillatum*	1
Pottiales	Pottiaceae	*Geheebia*	*Geheebiafallax*	5
Pottiales	Pottiaceae	*Geheebia*	*Geheebiaferruginea*	1
Pottiales	Pottiaceae	*Geheebia*	*Geheebiaspadicea*	1
Pottiales	Pottiaceae	*Gymnostomum*	*Gymnostomumaeruginosum*	3
Pottiales	Pottiaceae	*Microbryum*	*Microbryumdavallianum*	2
Pottiales	Pottiaceae	*Tortella*	*Tortellatortuosa*	3
Pottiales	Pottiaceae	*Tortula*	*Tortulaacaulon*	13
Pottiales	Pottiaceae	*Tortula*	*Tortulaacaulon*	1
Pottiales	Pottiaceae	*Tortula*	*Tortulacaucasica*	3
Pottiales	Pottiaceae	*Tortula*	*Tortulalindbergii*	3
Pottiales	Pottiaceae	*Tortula*	*Tortulamuralis*	5
Pottiales	Pottiaceae	*Tortula*	*Tortulamuralis*	6
Pottiales	Pottiaceae	*Tortula*	*Tortulaprotobryoides*	1
Pottiales	Pottiaceae	*Tortula*	*Tortulatruncata*	4
Scouleriales	Flexitrichaceae	*Flexitrichum*	*Flexitrichumflexicaule*	2
